# RETRACTION: Acetaldehyde Induces Neurotoxicity In Vitro via Oxidative Stress‐ and Ca^2+^ Imbalance‐Mediated Endoplasmic Reticulum Stress

**DOI:** 10.1155/omcl/9787694

**Published:** 2026-07-01

**Authors:** Oxidative Medicine and Cellular Longevity

RETRACTION: J. Cui, Y. Liu, X. Chang, et al., “Acetaldehyde Induces Neurotoxicity In Vitro via Oxidative Stress‐ and Ca^2+^ Imbalance‐Mediated Endoplasmic Reticulum Stress,” *Oxidative Medicine and Cellular Longevity*, no. 2019 (2019). https://doi.org/10.1155/2019/2593742.

The above article, published online on 9 January 2019 in Wiley Online Library (wileyonlinelibrary.com), has been retracted following the agreement the Chief Editor of the Journal Jeannette Vasquez‐Vivar; and John Wiley & Sons Ltd.

The retraction has been agreed following an investigation of the concerns raised by *Actinopolyspora biskrensis* on PubPeer [1].

More specifically, the investigation has identified unexpected similarities the HT22 cells treated with 4‐PBA in Figure 5b, and the control cells in Figure 6a.

The authors have been contacted to clarify these concerns and provide the raw data, however they were unable to do so.

As a result of the investigation, the data and conclusions of this article are considered unreliable.

The authors disagree with this retraction.
